# Photoelectrochemical Behavior of WO_3_ in an Aqueous Methanesulfonic Acid Electrolyte

**DOI:** 10.1021/acsphyschemau.2c00009

**Published:** 2022-03-28

**Authors:** Katarzyna Jakubow-Piotrowska, Dominik Kurzydlowski, Piotr Wrobel, Jan Augustynski

**Affiliations:** † Centre of New Technologies, University of Warsaw, S. Banacha 2c, 02-097 Warsaw, Poland; ‡ Faculty of Mathematics and Natural Sciences, Cardinal Stefan Wyszynski University in Warsaw, 01-038 Warsaw, Poland; § Faculty of Physics, University of Warsaw, Pasteura 5, 02-093 Warsaw, Poland

**Keywords:** photoelectrochemistry, mesoporous tungsten trioxide, WO_3_ photoanode, efficient water splitting, methanesulfonic acid, supporting electrolyte

## Abstract

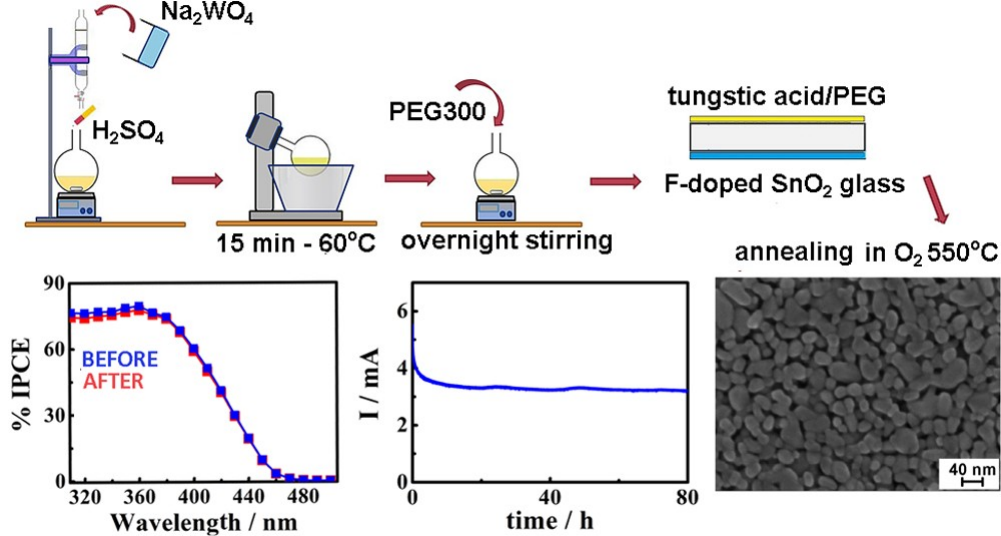

N-type semiconducting WO_3_ is widely investigated as a photoanode operating in water and seawater splitting devices. Because of the propensity of WO_3_ to favor photo-oxidation of acidic electrolyte anions and, in parallel, the formation on the electrode surface of the peroxo species, the choice of the appropriate electrolyte to allow stable operation of the photoanode is of critical importance. Our results from structural and photoelectrochemical tests performed using mesoporous WO_3_ photoanodes exposed to 80 h long photoelectrolysis in a 1 M aq. methanesulfonic acid supporting electrolyte demonstrate the photostability of both the WO_3_ photomaterial and the CH_3_SO_3_H electrolyte. The reasons for the stability of aqueous solutions of CH_3_SO_3_H are discussed on the basis of earlier literature reports.

## Introduction

Due to its optoelectronic properties, tungsten trioxide, WO_3_, is a multifunctional material with applications extending from photocatalysis^1−3^ to electrochromic^4^ and photochromic devices.^5,6^ With a band gap of ∼2.6 eV, WO_3_ absorbs photons in the blue part of the visible spectral range that enables it to deliver significant anodic photocurrents. Large photocurrent efficiencies observed for nanostructured WO_3_ photoanodes rely on relatively long hole diffusion length (∼150 nm)^1,7^ that may prevent bulk electron–hole recombination within the nanoparticles that form the film. At the same time, the efficient photogenerated current collection at the electrode substrate is favored by relatively large (in the range of 6.5–12 cm^2^ V^–1^ s^–1^) electron mobility in the crystalline WO_3_.^8,9^

The potential of the higher valence band (VB) edge located close to 2.9 V versus reversible hydrogen electrode (RHE)^10^ makes WO_3_ a powerful photocatalyst able to oxidize a wide range of compounds including most of the organic contaminants present in wastewater. Photoelectrolysis employing a WO_3_ photoanode, including extensively investigated water splitting,^2,3^ must be conducted in acidic (including strong acids) aqueous solutions of pH not higher than 4 to avoid the oxide dissolution occurring already in slightly alkaline solutions.

Among recent literature reports regarding applications of tungsten trioxide, WO_3_, as a photocatalyst and, in particular, as a photoelectrode, we noticed an article, published in this Journal, authored by Knoppel et al. dealing with photocorrosion of WO_3_ employed as photoanode in a series of electrolytes.^11^ Although the issue is of evident interest, we were surprised by the experimental procedure employed by the latter authors.^11^ In fact, their approach was based on very short (∼200 s long) experiments over which the WO_3_ photoanode dissolution was monitored using an inductively coupled plasma mass spectrometer (ICP-MS) coupled to a scanning flow photoelectrochemical, PEC, cell. In this context, we were astonished by the reported observation that the highest extent of dissolution occurred when the WO_3_ photoanode operated in a methanesulfonic acid electrolyte.^11^ Actually, according to our observations, CH_3_SO_3_H is the supporting electrolyte in which the WO_3_ photoanode reaches the highest steady water splitting photocurrents^12−16^ apparently without a deactivation known to occur in several other acidic electrolytes, associated with the formation of the surface peroxo species.^17,18^

Given the particular importance of the CH_3_SO_3_H supporting electrolyte for efficient water splitting at the WO_3_ photoanode,^12^ we undertook a series of new experiments intended to test the long-term stability of the photomaterial in the course of water photoelectrolysis. For those experiments, we synthesized relatively thin (∼1.2 μm thick) mesoporous film WO_3_ electrodes that, according to the predictions formulated by Knoppel et al.,^11^ should totally dissolve within a few hours when operating as photoanodes under UV–visible illumination in a 0.1 M aq. CH_3_SO_3_H electrolyte.

Herein, we report a detailed description of the WO_3_ films synthesis via the sol–gel method developed earlier in the author’s laboratory^19^ and their morphological, structural, and PEC characterization before, during, and following the photostability tests.

## Results and Discussion

### Preparation and Characterization of the WO_3_ Films

**Scheme 1:**
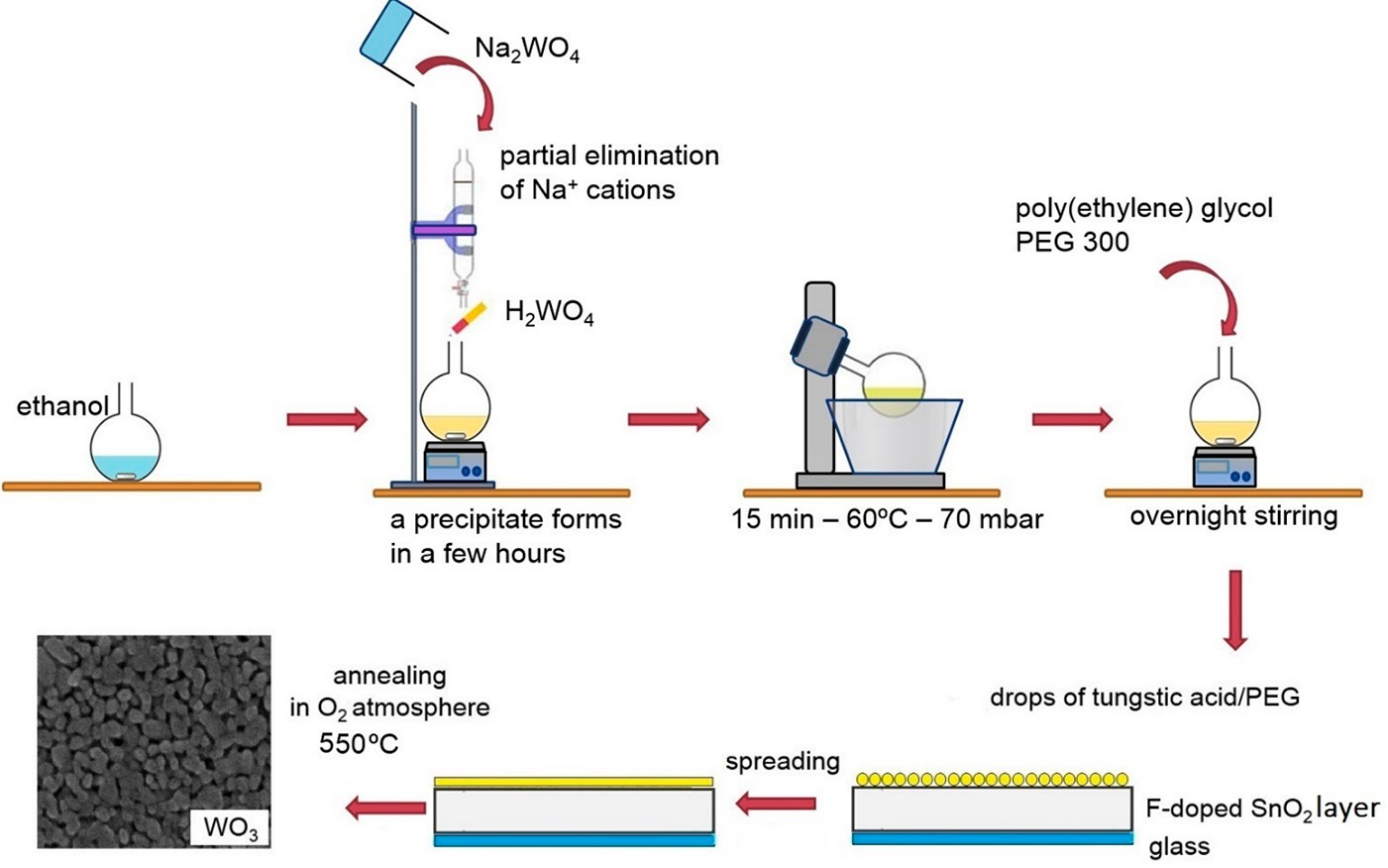
Chemical Precursor to WO_3_ Films by an Adapted Sol–Gel Method Preparation of the WO_3_ Layers

The F-doped SnO_2_ (FTO) coated glass sheets used as WO_3_ electrode substrates were cut into 1.5 × 3 cm^2^ slides and cleaned through successive sonication in soap water, water, and acetone before use. The WO_3_ films were obtained using a sol–gel method based on a fresh tungstic acid prepared by the elution of a 0.5 M aqueous solution of Na_2_WO_4_ through a proton exchange resin: Dowex 50 WX2-200. To retard the condensation of tungstic acid to form polyoxoanions, the solution was collected in ethanol. Upon completion of the elution, the solution was reduced in vacuum to a concentration of ∼0.5 M before the addition of low molecular weight polyethylene glycol (PEG) 300 and kept under continuous stirring. The used WO_3_/PEG ratio in the precursor was 0.4 w/w. The films were formed by a sequential layer-by-layer deposition/annealing method. The formation of a complex between tungstic acid and the hydrophilic polyethylene glycol delays the formation of fully crystallized monoclinic tungsten trioxide until a temperature of ∼500 °C.^19^ The precursor solution was coated onto the FTO substrates via doctor-blading and briefly air-dried, and then the films were annealed in flowing oxygen at 550 °C for 30 min. The WO_3_ films preparation process is represented in Scheme 1.

**Figure 1:**
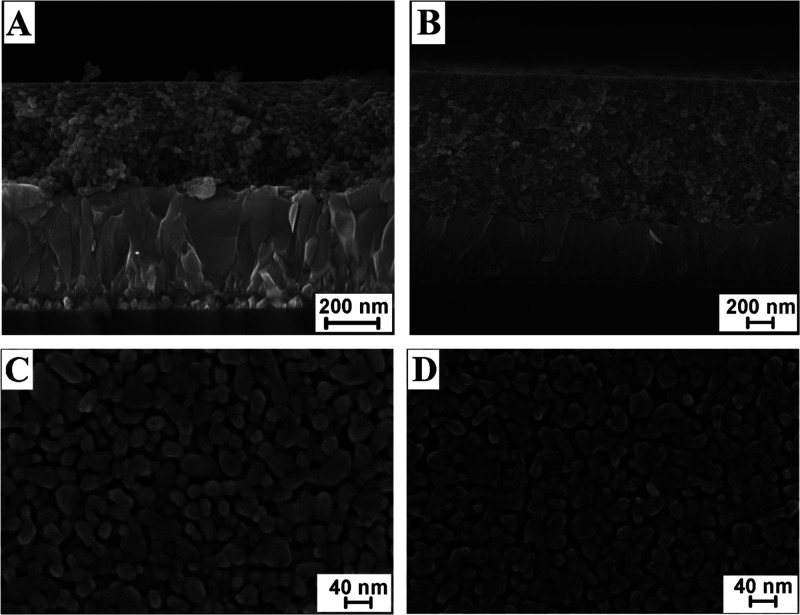
Cross-sectional SEM images of ∼0.4 μm (A) and 1.2 μm (B) thick WO_3_ films formed by an aqueous sol–gel method on F-SnO_2_/glass (FTO) substrates. Top-view SEM images of the WO_3_ films deposited on FTO were taken before (C) and after (D) the 80 h long photostability test.

A single standard application of the precursor typically produced an ∼0.4 μm thick WO_3_ film (Figure 1A). The WO_3_ films used in this work were formed by three consecutive applications of the precursor solution, each followed by the annealing in O_2_ at 550 °C for 30 min. The final thickness of such films (determined via cross-sectional scanning electron microscopy (SEM) imaging, shown in Figure 1B) was close to 1.2 μm. A SEM micrograph in Figure 1C shows the typical morphology of a WO_3_ film after heat treatment at 550 °C. The mesoporous film consists of a network of partly fused platelike particles with sizes of individual nanoparticles (NPs) in the range of 20–40 nm.

**Figure 2:**
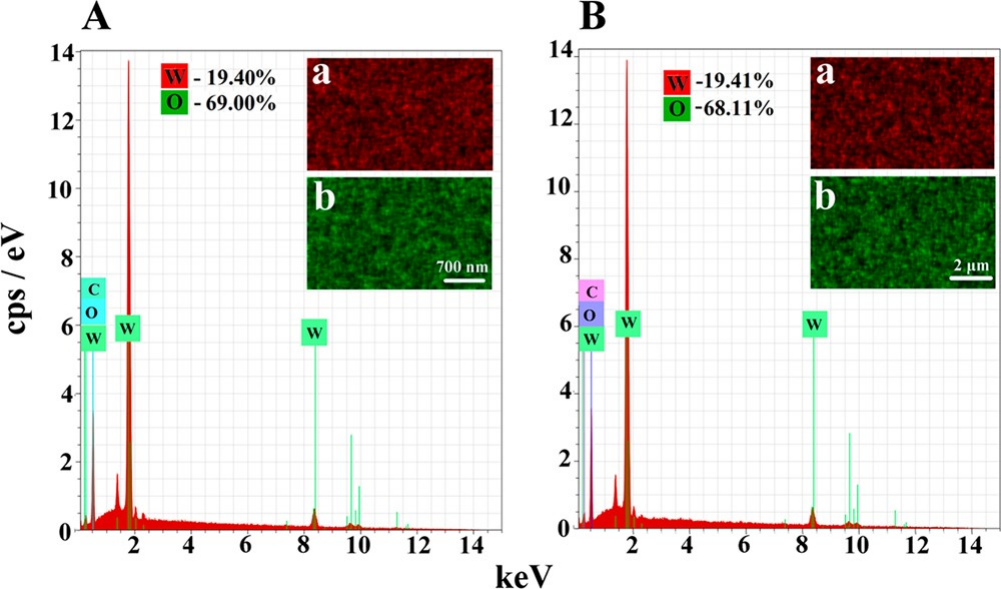
EDS analysis of a WO_3_ film deposited on FTO taken before (A) and after (B) the photostability test. Corresponding elemental mapping for tungsten (a) and oxygen (b).

The energy dispersive X-ray spectroscopy (EDS) analysis performed for the as-prepared WO_3_ film and after the sample was used for over 80 h as a photoanode (Figure 2) did not show any changes in the elemental film content.

**Figure 3:**
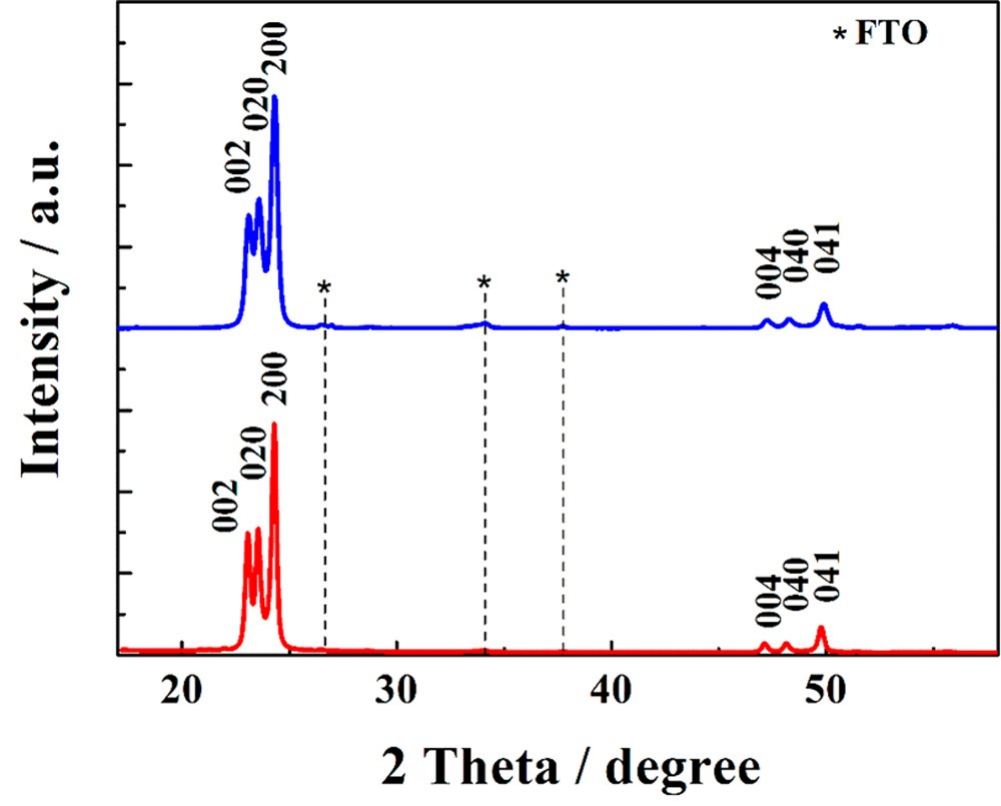
X-ray diffraction pattern of a WO_3_@FTO film annealed at 550 °C measured before (blue trace) and after (red trace) the 80 h long photostability test.

Although annealing at 550 °C markedly develops the film porosity, it preserves the monoclinic film structure (cf. Figure 3) characterized by three dominant X-ray diffraction (XRD) (002), (020), (200) peaks accompanied by less intense (004), (040), and (041) peaks indicating the preferential orientation of the WO_3_ crystallites parallel to the substrate (COD, Crystallography Open Database, # 96-210-6383).

**Figure 4:**
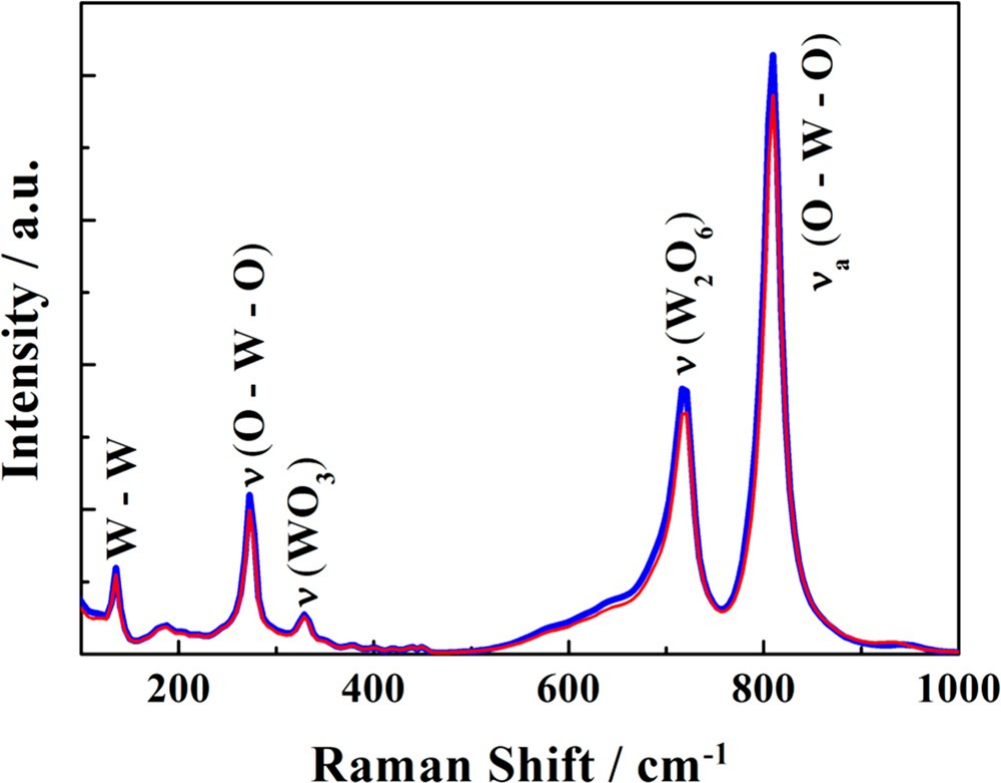
Raman spectra of a WO_3_ film deposited on FTO taken before (blue trace) and after (red trace) the photostability test.

The Raman spectrum shown in Figure 4 also displays the main features typical of the monoclinic structure of WO_3_ with prominent bands at ∼715 and 805 cm^–1^ and weaker bands at around 270 and 325 cm^–1^.

**Figure 5:**
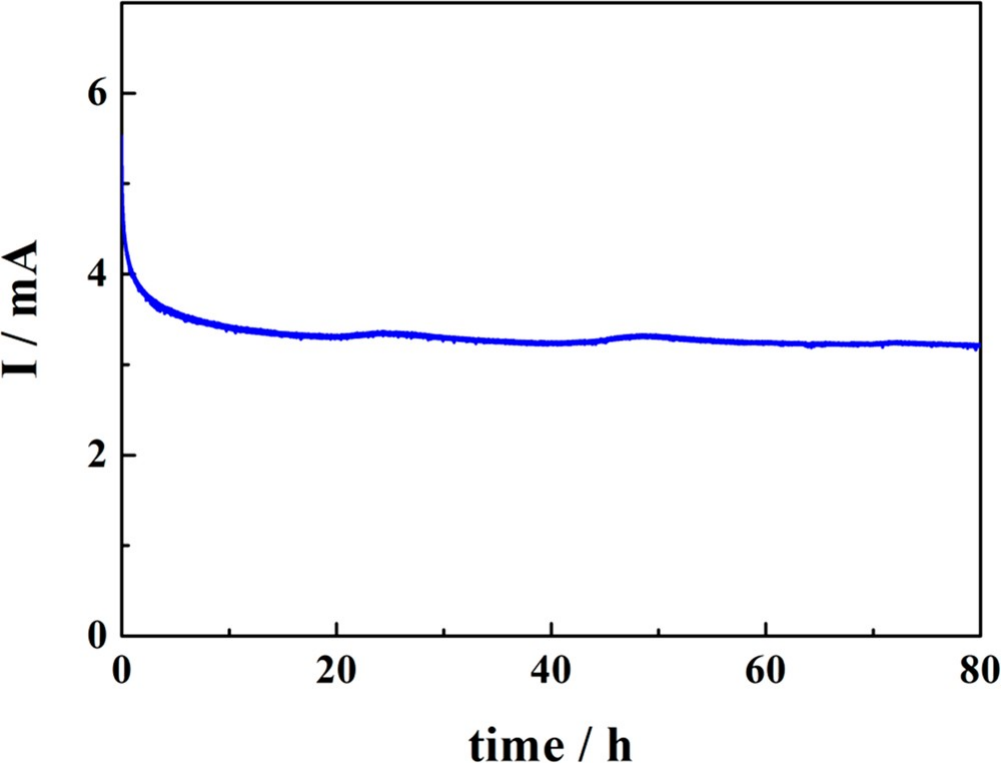
Plot showing the 80 h long stability test results for photocurrent vs time for an ∼1.2 μm thick WO_3_@FTO photoanode illuminated with AM 1.5G light and polarized at 1.2 V vs RHE in 1 M aqueous CH_3_SO_3_H solution.

We performed the PEC WO_3_ photostability tests using a large volume cell filled with 1 M aq. CH_3_SO_3_H electrolyte in which anodic and cathodic compartments were separated by a Nafion membrane. Figure 5 shows the evolution of the photocurrent monitored over a continuous 80 h long operation of the photoanode polarized at 1 V vs Ag/AgCl (∼1.2 V vs reversible hydrogen electrode, RHE) under simulated AM 1.5G (100 mW cm^–2^) sunlight. After an initial drop of the photocurrent attributable to filling the pores within the WO_3_ film by the evolved oxygen, the PEC performance of the photoanode remained stable along the entire electrolysis run.

**Figure 6:**
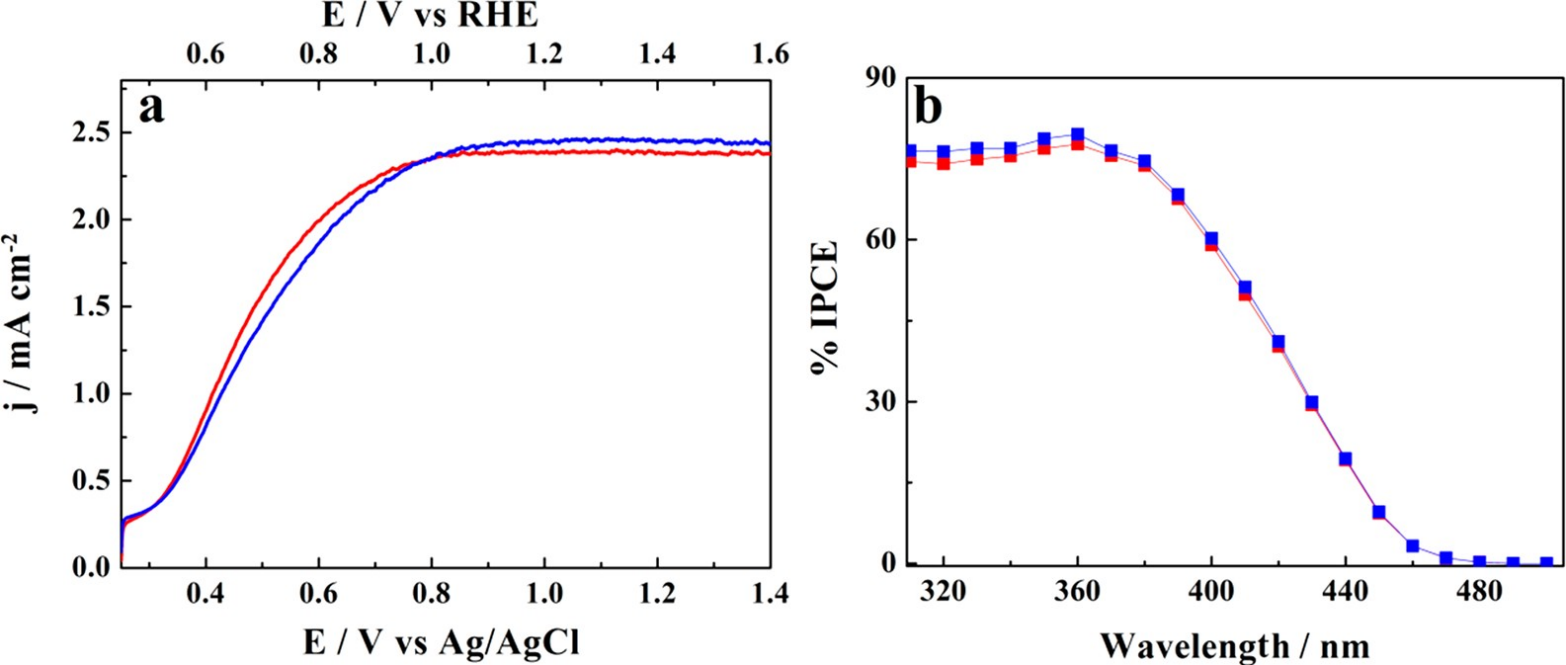
(a) Photoanodic current vs potential (*j*–*E*) plots for a WO_3_ electrode (∼1.2 μm thick) recorded in 1 M aqueous CH_3_SO_3_H electrolyte under simulated solar AM 1.5G (100 mW cm^–2^) irradiation. (b) Corresponding IPCE plots before (blue line) and after (red line) the 80 h long stability test.

Representative photocurrent–potential (*j*–*E*) characteristics for the WO_3_ photoanodes measured within a relatively small volume cell containing 40 mL of 1 M aq. CH_3_SO_3_H electrolyte can be seen in Figure 6a. The potential imposed to the WO_3_ electrode was swept from ∼0.25 V vs Ag/AgCl (i.e., 0.45 V vs RHE) corresponding to the open circuit, to 1.4 V. Under simulated solar AM 1.5G (100 mW cm^–2^) irradiation, the as-prepared photoanode reaches a plateau of 2.4 mA cm^–2^ at a potential of around 0.9 V_Ag/AgCl_ above which the photocurrent remained only limited by photon absorption (Figure 6a, blue line). A similar measurement was also performed for the WO_3_ photoanode, submitted earlier to the 80 h long stability test, and subsequently replaced in a cell containing 40 mL of the fresh 1 M aq. CH_3_SO_3_H electrolyte (cf. red trace in Figure 6a). Note that both plots are practically superimposed. A similar result was obtained when comparing the incident photon-to-current conversion efficiency (IPCE) spectra obtained for both electrodes, represented in Figure 6b. The latter result is particularly important since, given the indirect nature of the optical transition in WO_3_, the IPCEs close to the band edge are particularly sensitive to any changes in the film thickness. In fact, as shown earlier,^14,16^ the PEC performance of a WO_3_ photoanode is strongly affected by its film thickness that translates to the optical thickness. Consequently, for thicker (3 μm) WO_3_ films, the maximum IPCE appears already in the 390–400 nm wavelength range and measurable IPCEs are still observed up to 490–500 nm.^16^

It is important to recall that the excellent photostability of WO_3_ photoanodes in the aq. CH_3_SO_3_H electrolyte revealed by the PEC tests is entirely consistent with the fact that their structural characteristics (displayed in Figures 1C,D and 2–4) also remained unchanged after the prolonged photoelectrolysis run.

To complement the above results regarding the photostability of the WO_3_ photoanode, let us recall that also the stability of the 1 M aq. CH_3_SO_3_H water splitting electrolyte itself had been already clearly confirmed after a 3 day long continuous photoelectrolysis conducted under high intensity near-UV light.^12^ In fact, the Raman analyses of the electrolyte did not show any new features that would suggest that a transformation of the aq. CH_3_SO_3_H solution had occurred over prolonged photoelectrolysis.

Concomitant with our earlier results regarding the stability of the 1 M aq. CH_3_SO_3_H electrolyte^12^ are the literature reports specifying anhydrous conditions under which the CH_3_SO_3_^–^ anions may be oxidized to form bismethylsulfonyl peroxide
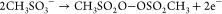
Thus, (CH_3_SO_3_)_2_ was produced through PEC oxidation at a WO_3_ photoanode in contact with the solutions of tetra(*n*-butyl)ammonium methanesulfonate in aprotic solvents, acetonitrile, and propylene carbonate.^20^ An earlier work had described the synthesis of a (CH_3_SO_3_)_2_ solid precipitate by electrochemical oxidation at a Pt electrode of a solution of CH_3_SO_3_Na in an anhydrous CH_3_SO_3_H.^21^ Interestingly, a former experiment performed in a 10 M aqueous solution of CH_3_SO_3_H led to the formation, with lower Faradaic efficiency, of an insoluble (CH_3_SO_3_)_2_ deposit on the Pt anode accompanied by local heating and explosive decomposition of the peroxide.^22^ These observations suggest that the CH_3_SO_3_^•^ radical intermediate is unstable in an aqueous environment. If the PEC oxidation of the CH_3_SO_3_^–^ anions from 1 M aq. methanesulfonic acid involves the formation of CH_3_SO_3_^•^ radical intermediates on the WO_3_ surface, presumably they react rapidly with water molecules to form oxygen. Although the formation of H_2_O_2_ as an intermediate cannot be completely excluded, the absence of the WO_3_ electrode passivation that is otherwise observed in HClO_4_ and HNO_3_ electrolytes (cf. Figure 1S, Supporting Information) and might be attributed to the formation of peroxo species,^17,18^ makes in the case of aq. CH_3_SO_3_H this hypothesis less probable. The results represented in Figure 1S, Supporting Information also show that the real issue affecting the photostability of the WO_3_ photoanode is most probably the formation of the peroxo species leading to a dramatic drop of the photocurrents generated along water splitting runs.

It seems interesting to recall in this connection that, in contrast with the case of methanesulfonic acid electrolyte, Raman analyses following a similar long photoelectrolysis experiment that involved 3 M aq. sulfuric acid had revealed formation at the WO_3_ photoanode of peroxodisulfates with about 80% Faradaic efficiency^17,12^ accompanied by a relatively slow electrode deactivation. Consequently, the PEC behavior of the WO_3_ electrodes in the two aq. CH_3_SO_3_H and H_2_SO_4_ electrolytes appears as totally different. Considering specifically the aspect of photocurrent stability, there is, however, a similarity between the behavior of the WO_3_ electrode in the aq. CH_3_SO_3_H electrolyte and that observed in acidic chloride solutions where photoelectrolysis produces a mixture chlorine and oxygen.^16−18,23^

## Conclusion

In summary, the performed long-term PEC tests demonstrated excellent photostability of mesoporous WO_3_ photoanodes in contact with a 1 M methanesulfonic acid used as the supporting electrolyte in water splitting experiments. These results parallel our earlier Raman spectroscopic measurements^12^ that also revealed the absence in the electrolyte of any new features that might be attributable to the photo-oxidation of CH_3_SO_3_H itself. With reference to the paper by Knoppel et al.^11^ that prompted our investigation reported herein, we wish to stress the requirement of following systematic reproducible experimental procedures prior to formulating categorical statements, like that regarding the instability of the WO_3_ photoanodes in the methanesulfonic acid electrolyte, that might create unwanted confusion. This concerns, in particular, the preparation method of WO_3_ films based on peroxo-tungstic acid known for being problematic and poorly reproducible.^18^

## Experimental Section

### Materials

Sodium tungstate dihydrate, polyethylene glycol (PEG) 300 used to prepare WO_3_ films, and methanesulfonic acid (99.5%) were purchased from Sigma-Aldrich. FTO-coated glass sheets with a resistance of 7 Ω/square (Sigma-Aldrich) served as electrode substrates. The solutions used for electrochemical measurements were prepared with Milli-Q water.

### Structural Characterization

SEM imaging of WO_3_ electrodes was performed using a Carl Zeiss Sigma HV workstation. The microscope was equipped with a Gemini electron column featuring in-lens secondary electron and backscattered electron detectors. EDS measurments were performed using XFlash 6I10 system, Bruker, equipped with SEM.

X-ray powder diffraction (XRD) measurements were performed on the as-grown samples using a Siemens D500 diffractometer equipped with a high-resolution semiconductor Si:Li detector using Cu Kα radiation (*U* = 40 kV, *I* = 30 mA). The powder diffraction patterns were measured in the θ/2θ scanning mode with a step of 0.02° and counting time of 10 s by step.

Raman spectra were acquired with the Alpha 300M+ confocal microscope (Witec Gmbh) equipped with a motorized stage. We used a 532 nm laser line delivered to the microscope through a single-mode optical fiber. The laser power at the sample did not exceed 7 mW. The backscattered Raman signal was collected through a 50× long working distance objective (NA = 0.42) and passed through a photonic optical beam to a lens based spectrometer (Witec UHTS 300, f/4 aperture, focal length 300 mm) coupled with a back-illuminated Andor iDUS 401 detector thermoelectrically cooled to −60 °C. The spectra were collected in the range of Raman shifts from 100 to 1000 cm^–1^ with the use of an 600 mm grating. For each sample, Raman mapping was conducted on a 50 × 50 μm^2^ area with a 1 μm step (totaling to 2 500 spectra) and a 0.3 s acquisition time. The spectra were postprocessed (background subtraction and cosmic-ray removal) with the Project FIVE software (Witec Gmbh). For all samples, the spectra in the mapped area were homogeneous. Consequently, an average over the 2500 spectra was made for each sample resulting in the spectra displayed in Figure 4.

### Photoelectrochemical Measurements

The photocurrent versus potential (*j*–*E*) plots and incident photon-to-current conversion efficiencies (IPCEs) were measured in a two-compartment Teflon cell, filled with ∼40 mL of 1 M aq. CH_3_SO_3_H electrolyte, equipped with a quartz window. The measurements were performed in a three-electrode configuration, with a platinum grid counter electrode and silver/silver chloride in sat. KCl (E = 0. 197 V vs SHE) as a reference electrode.

To obtain the *j*–*E* plots, the potential of the WO_3_ electrode (exposed surface area 0.28 cm^2^) was swept from the open-circuit potential (∼0.25 V vs Ag/AgCl) to 1.4 V at a rate of 10 mV/s using a CHI 660D electrochemical workstation. The simulated sunlight (AM 1.5G (100 mW cm^–2^) was provided by an Oriel 150 W solar simulator. The IPCEs of the photoanodes were determined using light from a 150 W xenon lamp passing through a photoelectric spectrometer (Instytut Fotonowy) featuring monochromator with a bandwidth of 10 nm. The absolute light intensity was measured with a model OL 730-5C UV-enhanced silicon detector (Gooch & Housego).

For monitoring the long-term WO_3_ photostability, we used a large volume quartz cell with anodic and cathodic compartments separated by a Nafion membrane, each containing 400 mL of a 1 M aq. CH_3_SO_3_H electrolyte. In that case, the total surface area of the deposited WO_3_@FTO (∼1.8 cm^2^) photoanode was exposed to the electrolyte.

## Supporting Information Available

Photocurrent versus time characteristics of the WO_3_ photoanodes recorded over prolonged electrolysis conducted in three different acid electrolytes (PDF)
