# Supplementary material for: Photoelectrochemical Behavior of WO3 in an Aqueous Methanesulfonic Acid Electrolyte
Source: ACS Phys Chem Au. 2022 Mar 28;2(4):299–304. doi: 10.1021/acsphyschemau.2c00009 (PMC9955116; doi:10.1021/acsphyschemau.2c00009)
Supplement: Supplementary file 1 — pg2c00009_si_001.pdf [file pg2c00009_si_001.pdf]

Supporting Information

"Photoelectrochemical Behavior of WO_3 in an Aqueous Methanesulfonic Acid Electrolyte."

Katarzyna Jakubow-Piotrowska[1], Dominik Kurzydlowski[2], Piotr Wrobel[3], Jan Augustynski[1]*

[1] Centre of New Technologies, University of Warsaw, S. Banacha 2c, 02-097, Warsaw, Poland.

[2] Faculty of Mathematics and Natural Sciences, Cardinal Stefan Wyszynski University in Warsaw, 01-038 Warsaw, Poland.

[3] Faculty of Physics, University of Warsaw, Pasteura 5, 02-093 Warsaw, Poland.

*Prof. J. Augustynski
E-mail: jan.augustynski@unige.ch
Centre of New Technologies, University of Warsaw, S. Banacha 2c, 02-097, Warsaw, Poland

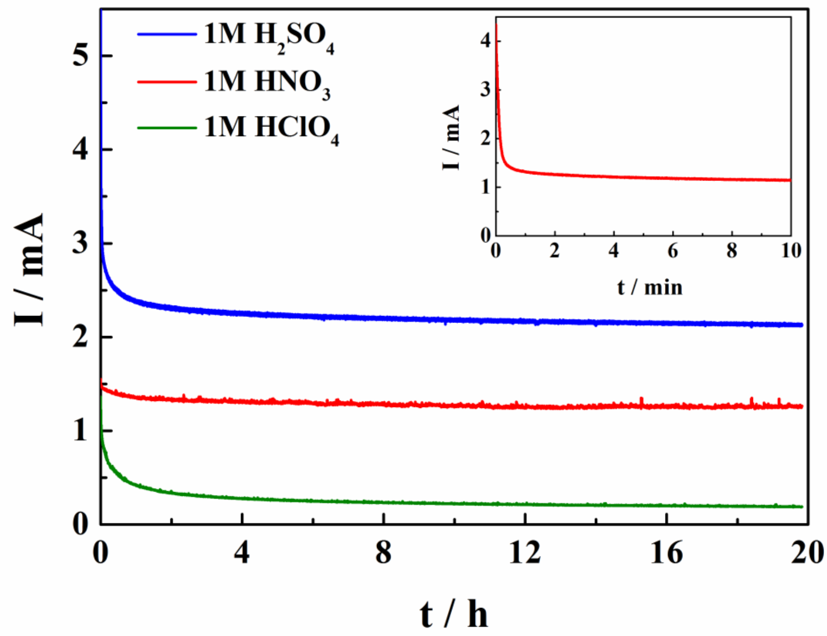

Figure S1. 20 h - long photocurrent vs time plots for a ca 1.2 µm-thick WO_3@FTO photoanode illuminated with AM 1.5G light and polarized at 1.2 V vs RHE in 3 different aqueous acid electrolytes. In response to a question raised by a Reviewer of the Ms., in the inset we show the rapid drop of the photocurrent recorded in the initial stage of the WO_3 electrode polarization in an aq. HNO_3 solution.
